# Ferroptosis-Mediated Cell-Specific Damage: Molecular Cascades and Therapeutic Breakthroughs in Diabetic Retinopathy

**DOI:** 10.3390/antiox15010001

**Published:** 2025-12-19

**Authors:** Yan Chen, Rongyu Wang, Nannan Zhang, Liangzhi Xu

**Affiliations:** 1 Reproductive Endocrinology and Regulation Laboratory, West China Second University Hospital, Sichuan University, Chengdu 610064, China; chenyan007@scu.edu.cn (Y.C.); rongyu-wang@scu.edu.cn (R.W.); 2 Department of Obstetrics and Gynecology, West China Second University Hospital, Sichuan University, Chengdu 610064, China; 3 Key Laboratory of Birth Defects and Related Diseases of Women and Children, Ministry of Education, Sichuan University, Chengdu 610041, China; 4 Department of Traditional Chinese Medicine, West China Second University Hospital, Sichuan University, Chengdu 610064, China; 5 National Center for Birth Defect Monitoring, West China Second University Hospital, Sichuan University, Chengdu 610064, China

**Keywords:** diabetic retinopathy, ferroptosis, GPX4, retinal microvascular endothelial cells, retinal pigment epithelial, photoreceptor cells

## Abstract

Diabetic retinopathy (DR), a leading cause of vision loss in diabetic patients, involves complex pathological mechanisms including neurodegeneration, microvascular damage, inflammation, and oxidative stress. Recent studies have identified ferroptosis—a ferrodependent cell death mechanism—as playing a pivotal role in DR development. Existing evidence indicates that oxidative stress and mitochondrial dysfunction induced by hyperglycemia may contribute to retinal damage through the ferroptosis pathway in DR. Ferroptosis inhibitors such as Ferostatin-1 have demonstrated protective effects against DR in animal models. The core mechanisms of ferroptosis involve iron homeostasis imbalance and lipid peroxidation, with key regulatory pathways including GPX4-dependent and non-dependent mechanisms (such as FSP1-CoQ10). Within the signaling network, Nrf2 inhibits ferroptosis, p53 promotes it, while Hippo/YAP functions are environment-dependent. Non-coding RNAs and epigenetic modifications (e.g., DNA methylation and histone modifications) also participate in regulation. In DR, iron overload, GPX4 dysfunction, and p53 upregulation collectively induce ferroptosis in various types of retinal cells, making these pathways potential therapeutic targets. This review not only elaborates the role of iron metabolism imbalance and ferroptosis pathway in the occurrence and development of DR but also summarizes the new therapeutic approaches of DR targeting ferroptosis pathway. Investigating the relationship between ferroptosis and DR not only helps unravel its core pathophysiological mechanisms but also provides theoretical foundations for developing novel therapeutic approaches.

## 1. Introduction

Diabetic retinopathy (DR), as one of the most common microvascular complications of diabetes mellitus (DM), is a leading cause of vision loss and blindness in working-age adults worldwide [1,2]. The prevalence of DR is on the rise globally. It is estimated that approximately 103.12 million DM patients worldwide were affected by DR in 2020, with projections indicating this number will increase to 129.84 million and 160.50 million by 2030 and 2045, respectively. This signifies that DR remains a pressing public health issue requiring urgent resolution [3]. Although DR can occur secondary to both type 1 and type 2 DM (T1DM and T2DM), its prevalence differs significantly between the two types. Specifically, the prevalence of DR is substantially higher in adults with T1DM (50.1%) than in those with T2DM (13.1%) [4,5]. The high-risk factors for DR include a long duration of DM, poor glycemic control (especially persistently elevated hemoglobin A1c levels) [6,7,8], concurrent dyslipidemia [9], obesity [10], concurrent dyslipidemia [9], coexisting hypertension [1,11], and the presence of diabetic nephropathy (DN) [12,13]. Certain genetic factors such as specific human leukocyte antigen genotypes [14], or particular genetic variants such as vascular endothelial growth factor (*VEGF*) and advanced glycation end receptor (*AGER*) genes are associated with an increased risk of DR [15]. Lifestyle factors, including sedentary behavior and particularly prolonged screen time, may elevate the risk of DR [16]. Conversely, the Mediterranean diet may have a beneficial and positive impact on the progression of retinal diseases [17].

The pathological mechanisms of DR are complex, involving multiple interrelated processes such as neurodegeneration, microvascular damage, inflammation, oxidative stress, and mitochondrial dysfunction [2,18]. Among them, retinal microangiopathy and neovascularization are the most characteristic pathological changes in DR, manifested as high blood sugar-induced dysfunction of retinal vascular endothelial cells, presenting as increased vascular permeability, microaneurysm formation, hemorrhage, hard exudates, and ultimately vascular occlusion and neovascularization. These newly formed blood vessels are prone to rupture and hemorrhage, leading to vitreous hemorrhage or tractional retinal detachment, which severely threatens vision [1,3]. Retinal vascular lesions are often accompanied by retinal neuronal degeneration [19]. Emerging research suggests that dysfunction of the neurovascular unit (NVU) may be the core pathological mechanism of DR. Retinal neurons, glial cells, and the vascular system collectively form the NVU. Under DM conditions, early NVU dysfunction—including neuronal death, glial cell activation, and blood-retinal barrier (BRB) disruption—lays the foundation for subsequent vascular pathologies [20]. It is also noticed that the involvement of immune cells and chronic inflammation serves as the core driving factor in the pathophysiology of DR [21,22,23].

Ferroptosis is a unique, regulated form of cell death characterized by the iron-dependent accumulation of lipid peroxides. It exhibits significant differences from other cell death modalities, such as apoptosis, necrosis, and autophagy, in terms of both morphological features and biochemical mechanisms [24,25,26]. The core mechanism of ferroptosis lies in iron metabolism imbalance, lipid peroxidation, and impaired antioxidant defense system [27]. In recent years, research on ferroptosis has experienced explosive growth, revealing that ferroptosis plays a critical role in maintaining cellular homeostasis and is closely associated with the occurrence and progression of various diseases, serving as both a causative factor and a therapeutic target. For instance, in neurodegenerative diseases such as Alzheimer’s disease, Parkinson’s disease, Huntington’s disease, and amyotrophic lateral sclerosis, common features include neuronal iron homeostasis imbalance, increased oxidative stress, lipid peroxidation, and elevated ferroptosis levels were wildly found [28,29]; In the field of oncology, many cancer cells exhibit sensitivity to ferroptosis, and inducing ferroptosis can inhibit tumor growth and enhance the efficacy of chemotherapy and radiotherapy [30,31]. In addition, ferroptosis plays a critical role in various diseases such as acute kidney injury, myocardial ischemia–reperfusion injury, organ fibrosis, degenerative bone and joint diseases (e.g., osteoarthritis, osteoporosis), and autoimmune diseases [32,33,34,35,36], and it also plays a crucial role in metabolic syndromes such as obesity, DM, insulin resistance, hyperlipidemia, hypertension, polycystic ovary syndrome, and metabolic associated fatty liver disease [37,38].

DM, as a complex chronic metabolic disease, has one of its core pathological hallmarks being the loss or dysfunction of functional pancreatic β-cells. High glucose levels, inflammatory factors, or DM-simulating agents such as streptozotocin (STZ) can induce ferroptosis in β-cells. Concurrently, the occurrence of ferroptosis leads to insufficient insulin secretion, exacerbating the progression of DM, suggesting that ferroptosis is involved in the pathological process of DM [39]. The fatal causes of DM are mostly related to its severe complications, which involve multiple organs throughout the body, including DR, DN, diabetic cardiomyopathy (DC), etc. Ferroptosis has been widely demonstrated in the pathophysiological processes of these complications [40,41,42]. It was discovered early on that under conditions of DM, iron accumulation in the retina is increased, suggesting that DR may be related to iron overload [43]. In recent years, it has been gradually recognized that ferroptosis, as an iron-dependent form of cell death, can lead to dysfunction of retinal microvascular endothelial cells (RMECs) and pathological changes in retinal pigment epithelial cells (RPECs), among other DR alterations [44,45]. Meanwhile, the ferroptosis inhibitor Ferrostatin-1 can effectively ameliorate tissue and cellular damage in DR [46]. The upregulation of thioredoxin-interacting protein (TXNIP) induced by the hyperglycemic state of DM and the associated oxidative stress lead to mitochondrial dysfunction, mitophagy, and ferritinophagy, subsequently causing the release of ions within cells and resulting in retinal iron overload, which may be the driving factor of ferroptosis in DR [47]. In this review, we will provide a detailed summary and elaboration on recent studies regarding ferroptosis and the role and mechanisms during DR occurrence and development within it. Additionally, we will introduce some potential DR treatment approaches targeting the ferroptosis pathway, aiming to offer researchers and clinicians more comprehensive research perspectives and therapeutic strategies for DR.

## 2. Mechanisms and Regulation of Ferroptosis

### 2.1. Iron Homeostasis Imbalance and Lipid Peroxidation

Iron is the core driving factor of ferroptosis. Intracellular iron homeostasis is strictly regulated to ensure normal cellular function while avoiding toxicity induced by iron overload. Cells uptake trivalent iron (Fe^3+^) through transferrin receptor 1 (TfR1), which is mediated by endocytosis. Within the endosome, Fe^3+^ is reduced to divalent iron (Fe^2+^) by six-transmembrane epithelial antigen of prostate 3 (STEAP3). The Fe^2+^ is then transported from the endosome into the cytoplasm via divalent metal transporter 1 (DMT1). Fe^2+^ primarily exists in two forms within the cell: bound iron (e.g., bound to ferritin) and free iron (also known as labile iron, referred to as the labile iron pool, LIP) [48]. The Fe^2+^ in LIP exhibits considerable instability and serves as a significant source of intracellular reactive oxygen species (ROS) generation, as LIP can directly participate in the Fenton reaction to produce highly reactive hydroxyl radicals (•OH), thereby driving lipid peroxidation and subsequently inducing ferroptosis [49]. The exporter of intracellular iron relies on two proteins: ferroportin (FPN) and ferritin. Of these, the activity of FPN is regulated by the iron-regulatory hormone hepcidin [50,51]. The iron-storage protein ferritin undergoes degradation and releases increased amounts of Fe^2+^ via nuclear receptor coactivator 4 (NCOA4)-mediated ferritinophagy, thereby exacerbating the accumulation of intracellular LIP. Meanwhile, elevated levels of hepcidin result in the internalization and degradation of FPN, which reduces iron export and consequently leads to further intracellular LIP accumulation [49].

The biosynthesis of iron metabolism-related proteins such as ferritin and FPN is regulated at the transcriptional level by nuclear factor erythroid 2-related factor 2 (Nrf2) and at the translational level by the Iron-Regulatory Protein (IRP)/Iron-Responsive Element (IRE) system. Nrf2 is a transcription factor capable of sensing cellular oxidative stress, which under steady-state conditions is constitutively expressed and rapidly degraded by the proteasome. Increased oxidative stress mediates the dissociation of Nrf2 from the complex responsible for its ubiquitination and degradation. Once released from this complex, Nrf2 translocates to the nucleus, where it possesses deoxyribonucleic acid (DNA)-binding sites and regulates over 200 genes [52]. Many of these genes play a role in antioxidant defense and redox balance, with some being crucial for iron homeostasis. These include heme oxygenase-1 (HO-1), subunits of FPN, and bone morphogenetic protein-6, which induces hepatic ferritin production in the liver [53]. IRP1 and IRP2 are cytoplasmic RNA-binding proteins that regulate cellular iron homeostasis by binding to IREs—stem-loop structures within the 5′ or 3′ untranslated regions (UTRs) of target mRNAs. IRPs are sensitive to cellular iron and oxygen concentrations. Their RNA-binding activity increases under low iron conditions, whereas IRP2 responds to elevated oxygen and reactive oxygen/nitrogen species (ROS/RNS) levels through increased degradation. In contrast, IRP1 requires high oxygen/ROS/RNS concentrations to transition from its initial aconitase form into an RNA-binding active protein conformation [54,55,56,57]. These findings suggest a close association between iron metabolism homeostasis and cellular redox status.

Lipid peroxidation is a hallmark of ferroptosis, particularly referring to the peroxidation of polyunsaturated fatty acids (PUFAs) and esterified forms like phospholipids. PUFAs in the cell membrane, especially arachidonic acid and adrenic acid, are initially activated by acyl-coenzyme A(CoA) synthetase long-chain family member 4 (ACSL4) to form acyl-CoA derivatives. These derivatives are subsequently incorporated into membrane phospholipids by lysophosphatidylcholine acyltransferase 3, resulting in the generation of phospholipid-bound PUFAs (PL-PUFAs) [58,59]. In LIP, ferrous ions catalyze hydrogen peroxide (H_2_O_2_) in the Fenton reaction to generate highly reactive •OH, which attack PL-PUFAs, initiating a chain reaction that produces a large amount of lipid hydroperoxides (PL-PUFAs-OOH). When these highly reactive lipid peroxides accumulate to a certain level, they cause structural damage and functional loss of the cell membrane, ultimately leading to cell death. This is the general process of ferroptosis [48]. In recent years, it has been discovered that, as a complement to the Fenton reaction, ROS generation mediated by nicotinamide adenine dinucleotide phosphate (NADPH) oxidase (NOX) can synergistically promote lipid peroxidation and ferroptosis. The NOX family is the sole enzyme in cells that directly catalyzes ROS production. It is localized on the cell membrane and transfers electrons provided by cytoplasmic NADPH across biological membranes, reducing oxygen to superoxide, thereby inducing lipid peroxidation of the cell membrane [60,61]. Currently, at least three members of the NOX family (NOX1, *CYBB*/NOX2, and NOX4) have been found to promote the process of ferroptosis through different regulatory mechanisms [62,63].

### 2.2. GPX4-Dependent Antioxidant Defense Failure

Cells have various antioxidant mechanisms to combat lipid peroxidation, with glutathione peroxidase 4 (GPX4) being the most crucial anti-ferroptosis enzyme. GPX4 employs glutathione (GSH) as a reductant to transform harmful lipid hydroperoxides (PL-PUFAs-OOH) into benign lipid alcohols (PL-PUFAs-OH), thus halting the propagation of lipid peroxidation. The preservation of intracellular GSH levels depends on System Xc^−^, a cystine/glutamate antiporter consisting of solute carrier family 7 member 11 (SLC7A11) and solute carrier family 3 member 2 (SLC3A2), which facilitates the transport of extracellular cystine into cells for GSH production. Inhibiting System Xc^−^ results in decreased cystine uptake, leading to a depletion of intracellular GSH. Consequently, GPX4 becomes impaired, making cells more vulnerable to lipid peroxidation and ultimately triggering ferroptosis [64]. Currently, three isoenzyme forms of GPX4 have been identified: cytosolic (cGPX4), mitochondrial (mGPX4), and nuclear (nGPX4), which exhibit distinct temporal-spacial expression patterns during embryonic development and adult life [65]. The expression and functional activity of GPX4 are also intricately regulated by multiple factors, which influence cellular sensitivity to ferroptosis and thereby determine disease onset and progression. For example, the ubiquitin-specific protease 8 modulates the homeostasis of GPX4, affecting cancer cells’ susceptibility to ferroptosis [66]. Furthermore, acid sphingomyelinase-dependent autophagic degradation of GPX4 is crucial for the execution of ferroptosis [67]. The deubiquitinase otubain 1 promotes tumor progression by stabilizing GPX4 and inhibiting ferroptosis in colorectal cancer [68]. Recently, more GPX4 regulatory factors have been identified. For instance, the latency-associated nuclear antigen encoded by kaposi sarcoma-associated herpesvirus can promote the susceptibility of HeLa cells to ferroptosis by suppressing *Nrf2*/GPX4 and upregulating mouse double minute 2 homolog [69]. Further, vitamin E, a well-known antioxidant, works synergistically with GPX4 to protect hematopoietic stem cells and progenitor cells from lipid peroxidation and ferroptosis [70].

### 2.3. GPX4-Independent Pathways in Regulation of Ferroptosis

#### 2.3.1. FSP1-CoQ10 Pathway

Ferroptosis suppressor protein 1 (FSP1) is an enzyme encoded by the *FSP1* gene that reduces ubiquinone (Coenzyme Q10, CoQ10) to form ubiquinol (the reduced form of coenzyme Q10, CoQ10H_2_), functioning independently of the GSH-GPX4 pathway as a key defense mechanism against ferroptosis [71]. FSP1 utilizes NADPH as an electron donor to catalyze the reduction of CoQ10 to CoQ10H_2_. The generated CoQ10H_2_ acts as a lipid-soluble antioxidant, directly capturing lipid radicals (such as lipid peroxyl radicals LOO•) and interrupting the lipid peroxidation chain reaction [71]. The N-terminal myristoylation modification of FSP1 enables its localization to the cell membrane, which is essential for the role in reducing CoQ10 within the membrane lipid compartment. Structural biology study reveals that the interaction between the flavin adenine dinucleotide-binding domain of and the NADPH-binding domain is crucial for catalytic efficiency of FSP1 [72]. Vitamin K can be reduced by FSP1 to its hydroquinone form, enhancing antioxidant capacity. Research indicates that the vitamin K cycle exhibits cross-complementarity with the FSP1-CoQ10 pathway, jointly maintaining membrane lipid homeostasis [73]. In various cancer cells (such as clear cell carcinoma of the kidney and prostate adenocarcinoma), FSP1 overexpression inhibits ferroptosis by maintaining CoQ10H_2_ levels, leading to chemotherapy resistance. Knocking out FSP1 can increase cancer cell sensitivity to erastin and RSL3 by 3–5 times [74,75].

#### 2.3.2. DHODH-CoQ10 Pathways

In addition to FSP1, the dihydroorotate dehydrogenase (DHODH) and CoQ10 constitute another crucial antioxidant defense system. By inhibiting mitochondrial lipid peroxidation, they serve as a key pathway regulating ferroptosis. DHODH is an enzyme located on the inner mitochondrial membrane and participates in the pyrimidine biosynthesis pathway. Its core function involves converting dihydroorotate to orotate while facilitating the reduction of CoQ10 to form CoQ10H2. This process relies on the mitochondrial electron transport chain to sustain the regenerative capacity of CoQ10H2 [76,77]. It is evident that DHODH, positioned on the inner mitochondrial membrane, and FSP1, located on the cell membrane, collectively establish a complementary dual defense antioxidant network within the cell. Mao et al. have shown that the knockout of *DHODH* elevates lipid peroxide levels, making cancer cells more susceptible to ferroptosis inducers, such as Erastin [76]. In tumors deficient in GPX4, DHODH inhibitors can selectively induce ferroptosis, indicating that DHODH also serves as an independent regulatory pathway for ferroptosis, separate from GPX4 [78]. In DM complications and liver injury, the DHODH-CoQ10 pathway maintains redox homeostasis and alleviates ferroptosis-driven tissue damage [42].

### 2.4. Key Signaling Regulatory Network in Ferroptosis

#### 2.4.1. Nrf2 Signaling Pathway

Nrf2 is a transcription factor which activates downstream gene expression by binding to antioxidant response elements (ARE) in response to cellular oxidative stress to maintain redox homeostasis [79]. Under ROS stimulation, the Kelch-like ECH-associated protein 1 (Keap1), an Nrf2 inhibitory protein undergoes conformational changes, releasing Nrf2 to facilitate its translocation into the nucleus and initiating ARE transcription, which represents the canonical mechanism of Nrf2 signaling activation [80]. In the regulation of ferroptosis, Nrf2 activates the transcription of key genes involved in GSH synthesis, such as glutamate-cysteine ligase modifier subunit, *SLC7A11*, and *GPX4*, thereby blocking the core execution mechanism of ferroptosis. Additionally, Nrf2 reduces intracellular free iron by upregulating iron metabolism-related genes, including ferritin heavy chain 1 (*FTH1*) and *FPN*, inhibiting the Fenton reaction. This decreases the accumulation of reactive ROS and lipid peroxidation, eliminating the initiating steps of ferroptosis. Furthermore, Nrf2 enhances the expression of fatty acid desaturase, particularly stearoyl-CoA desaturase 1 (*SCD-1*), reducing the proportion of PUFAs and thus lowering the substrate content for lipid peroxidation [81,82,83]. The Nrf2 signaling pathway is influenced by other classical signaling pathways. For instance, in the cerebral ischemia hypoxia model, Kellerin activates the phosphatidylinositol 3-kinase–Akt (PI3K-Akt) pathway in neurons. The activated Akt can enhance the stability of Nrf2 through phosphorylation, thereby exerting antioxidant and ferroptosis inhibitory effects [84]. In melanoma, the Ca^2+^/calmodulin-dependent protein kinase 2–Adenosine monophosphate-activated protein kinase (AMPK) signaling axis can also activate Nrf2, enhancing tumor cell resistance to ferroptosis [85]. Moreover, under hypoxic conditions, the accumulation of p62 competitively binds to Keap1, promoting the release of Nrf2 and reducing cellular sensitivity to ferroptosis [86]. Additionally, the BTB and CNC homology 1 protein can competitively bind to ARE with Nrf2, promoting the suppression of ferroptosis-related genes (such as *HO-1* and *SLC7A11*), thereby facilitating the occurrence of ferroptosis [87].

#### 2.4.2. p53 Pathway

The p53 signaling pathway exhibits multi-layered regulatory roles in ferroptosis, with mechanisms involving direct targeting of key ferroptosis molecules and regulation of iron metabolism among other pathways. Firstly, p53 transcriptionally represses the cystine/glutamate transporter *SLC7A11*, a component of system Xc^−^, thereby reducing intracellular cystine uptake and subsequently decreasing GSH synthesis [88]. In liver cancer cells, p53 directly binds to the *SLC7A11* promoter, downregulates its expression, leading to the accumulation of lipid peroxide, thereby promoting ferroptosis [89]. Arachidonic acid 12-lipoxygenase (*ALOX12*) is located on chromosome 17p13.1 (near the *p53* locus) and serves as an essential effector in p53-mediated ferroptosis. It catalyzes the lipid peroxidation of PUFAs, which can regulate ferroptosis independently of *GPX4* [90]. In vascular endothelial cells, angiotensin II (Ang-II) induces ferroptosis through the p53-ALOX12 axis, a process that is blocked by the p53 inhibitor Pifithrin-α [91]. Additionally, p53 activates *NCOA4*, mediates ferritinophagy, increases free iron levels, promotes ROS generation through the Fenton reaction, thereby facilitating ferroptosis [92]. p53 also regulates the expression of *DMT1* through signal transducer and activator of transcription 6 (*STAT6*), affecting intracellular iron uptake. The traditional Chinese medicine Maijitong inhibits *DMT1* by activating *STAT6*, thereby blocking p53-mediated ferroptosis and alleviating atherosclerosis [93]. Furthermore, *NOX1* can bind with dipeptidyl peptidase 4 under the regulation of p53 protein to mediate plasma membrane lipid peroxidation response, thereby promoting ferroptosis [62]. Recent studies have found that p53 can suppress the expression of vitamin K epoxide reductase complex subunit 1-like 1 (*VKORC1L1*) at both mRNA and protein levels. The latter protects cells from ferroptosis by generating reduced vitamin K, known as vitamin K hydroquinone, a process that is also independent of the GSH/GPX4 pathway [94].

#### 2.4.3. Hippo/YAP Pathway

The Hippo signaling pathway primarily senses mechanical cues from cell–cell contact, extracellular matrix stiffness, cell adhesion, and cell spreading. This pathway regulates the subcellular localization and activity of its downstream transcriptional regulators, Yes-associated protein (YAP) and transcriptional co-activator with PDZ-binding motif (TAZ), through a sequential kinase cascade mediated by Mammalian Ste20-like kinase 1/2 and large tumor suppressor kinase 1/2 (*LATS1/2*). When the Hippo pathway is activated, LATS1/2 phosphorylates YAP/TAZ, leading to their cytoplasmic retention and subsequent degradation, thereby suppressing transcriptional activity. Conversely, when the Hippo pathway is inactivated, YAP/TAZ translocates into the nucleus and binds to TEA DNA-binding domain transcription factors, driving the expression of target genes [95]. Research has found that cell density (response to intercellular contact state) regulates YAP/TAZ activity through the Hippo pathway, thereby determining cellular sensitivity to ferroptosis. When cells are at low density, the Hippo pathway is inhibited, subsequently activating YAP/TAZ transcriptional activity and promoting ferroptosis. Conversely, when cells are at high density, Hippo is activated, thereby suppressing YAP/TAZ activity and inhibiting ferroptosis. This explains why renal cancer cells exhibit a 4–7 times higher sensitivity to ferroptosis under low cell density conditions [96,97]. YAP/TAZ may influence ferroptosis by activating the transcription of certain ferroptosis regulatory genes. For example, as a component of System Xc^−^, the expression of *SLC7A11* in liver cancer cells is transcriptionally regulated by YAP, while aldo-keto reductase 1C3 increases the expression of *SLC7A11* by activating YAP, thereby regulating ferroptosis [98]. Furthermore, the YAP protein also promotes the ubiquitination and degradation of *SLC7A11* through the E3 ubiquitin ligase S-phase kinase-associated protein 2, inhibits cystine uptake, weakens GSH synthesis capacity, and subsequently promotes ferroptosis by suppressing the GPX4-dependent pathway [97]. In ovarian cancer, TAZ activation promotes the expression of Angiopoietin like protein 4, activates the NOX2 complex, increases ROS production, and enhances lipid peroxidation [63]. In low-density renal cancer cells, TAZ activates the expression of epithelial membrane protein 1, which subsequently increases the expression of *NOX4*, thereby promoting ferroptosis [96].

### 2.5. Epigenetics and Non-Coding RNA Regulation

#### 2.5.1. DNA Methylation

DNA methylation is an important epigenetic modification that regulates gene expression by adding a methyl group to the C5 position of cytosine, typically within CpG islands. Melanoma Antigen Family A 6 (*MAGEA6*), a member of the *MAGE* gene family, is scarcely expressed in normal tissues except for those related to reproduction, such as the testes and placenta. However, it is often overexpressed in various malignant tumors. As part of the E3 ubiquitin ligase complex, MAGEA6 binds with Tripartite Motif-containing 28 to target and degrade the tumor suppressor AMPKα1, thereby exhibiting strong pro-oncogenic effects [99]. In the pancreatic carcinoma model, high expression of *MAGEA6* significantly inhibited AMPK signaling-dependent macroautophagy, thereby promoting tumor progression [100]. Recent studies on acute myeloid leukemia have indicated that the expression of *MAGEA6* is regulated by DNA methylation. When tumor cells are treated with decitabine, the methylation levels in the MAGEA6 promoter region decrease, which results in elevated expression of this gene. Consequently, this leads to reduced AMPK activity and diminished activity of the AMPK signaling-dependent downstream *SLC7A11-GPX4* antioxidant system, thereby facilitating ferroptosis in the cells [101]. In liver cancer, the UHRF1 protein suppresses the expression of GSH-transferase zeta 1 (*GSTZ1*) by promoting DNA hypermethylation. *GSTZ1* plays a role in maintaining cellular GSH levels and regulating the redox balance. Its inhibition disrupts the intracellular glutathione redox state and the NADP^+^/NADPH balance, elevating lipid peroxidation and inducing ferroptosis [102].

#### 2.5.2. Post-Translational Modifications of Histones

Histone modifications, as essential epigenetic mechanisms, primarily function to regulate chromatin accessibility for transcriptional regulatory factors. Recent studies have revealed that histone modifications play a central role in various diseases by modulating the expression of ferroptosis-related genes and the activity of signaling pathways. Research has demonstrated that the repressive histone mark Histone H3 lysine 27 trimethylation is enriched in the promoter regions of key ferroptosis genes (such as *GPX4* and *SLC7A11*) in multiple cancer cell types, thereby suppressing their transcriptional expression and promoting ferroptosis in cancer cells [103,104]. Furthermore, as an activating marker, H3K4me3 promotes the transcription of ferroptosis-related genes (such as *ACSL4*), increases the synthesis of polyunsaturated fatty acid phospholipids (PUFA-PLs), and can also accelerate lipid peroxidation and cellular ferroptosis [104,105]. Recent study have identified that histone deacetylase inhibitors (HDACi) significantly increase the susceptibility of colorectal cancer to ferroptosis, indicating that the epigenetic modification mechanism of histone acetylation is involved in regulating ferroptosis [106]. The study found that HDACi specifically targets *HDAC1* and promotes the acetylation of histone H3 lysine 27 acetylation (H3K27ac) on the fat mass and obesity-associated gene (*FTO*) and AlkB homolog 5 (*ALKBH5*), thereby significantly activating the expression of *FTO* and *ALKBH5*. As two RNA demethylases, FTO and ALKBH5 reduce N6-methyladenosine (m6A) modification on *FSP1* mRNA, leading to the degradation of this transcript. This subsequently inhibits the FSP1-CoQ10 antioxidant pathway and promotes ferroptosis [106]. Furthermore, the *GPX4* gene is also directly regulated by histone methylation and acetylation, as increased Histone H3 lysine 4 trimethylation and H3K27ac marks in the promoter region of the *GPX4* gene have been observed in cancer cells, which correlates with elevated GPX4 expression and reduced cellular iron sensitivity [107].

Histone lactylation is a recently discovered epigenetic regulatory mechanism involving histone modification. Research has indicated that during lung injury, lactic acid can modulate the level of m6A level in alveolar epithelial cells. This occurs by facilitating p300-mediated lactylation of H3K18la and its interaction with the Methyltransferase-like 3 (*METTL3*) promoter region. *METTL3*-mediated m6A modification is concentrated in *ACSL4* and regulates its mRNA stability via a YT Homology Domain Containing 1-dependent pathway, which in turn leads to increased PL-PUFAs lipid peroxidation substrates and heightened ferroptosis sensitivity [108]. The increase in H3K18la also enhances the transcriptional activity of the mitochondrial protein Nitrogen fixation 1 (*NFS1*). *NFS1* and iron-sulfur cluster assembly enzyme suppress ferroptosis by increasing the biosynthesis of iron-sulfur clusters (Fe-S), thereby reducing the susceptibility of hepatocellular carcinoma to ferroptosis after incomplete microwave ablation [109].

#### 2.5.3. Non-Coding RNA

The regulatory role of non-coding RNA (ncRNA) in ferroptosis is a complex, multi- layered process involving multiple mechanisms, primarily achieved by influencing three core aspects: iron metabolism, lipid peroxidation, and the antioxidant system. For instance, in non-small cell lung cancer, lncRNA *NEAT1* upregulates *ACSL4* levels by adsorbing miR-362-3p, promoting the synthesis of polyunsaturated fatty acid phospholipids and increasing cellular susceptibility to ferroptosis [110]. Meanwhile, the circular RNA *circ0008035* alleviates the inhibition on the iron transport protein SLC38A1 by adsorbing miR-150-5p, thereby increasing intracellular iron load and consequently enhancing cellular sensitivity to ferroptosis [111]. In leukemia cells, lncRNA LINC00618 reduces the levels of lysine-specific histone demethylase 1 (*LSD1*) and *SLC7A11*, accelerates iron ion efflux, thereby inhibiting ferroptosis. Vincristine can induce the production of *LINC00618*, consequently increasing the sensitivity of leukemia cells to ferroptosis [112]. miR-324-3p has been found to directly target *GPX4* mRNA in various tumor cells, inhibiting the translation of *GPX4* and thereby reducing cellular antioxidant capacity [113,114,115].

The elaborate regulatory network of ferroptosis, encompassing iron homeostasis, lipid peroxidation, and multifaceted antioxidant defenses (both GPX4-dependent and independent), along with key signaling pathways and epigenetic regulation, establishes a comprehensive mechanistic framework. It is within this framework that we now examine the compelling evidence linking ferroptosis to DR. The diabetic retinal milieu, which is characterized by metabolic dysregulation, oxidative stress, and inflammation, creates conditions that directly engage these core ferroptotic pathways, primarily through driving iron overload and lipid peroxidation.

## 3. The Molecular Regulatory Network of Ferroptosis in DR

### 3.1. Iron Overload in the Retina of DR

Iron plays multiple roles in the normal physiological functions of the retina, including processes such as phototransduction, the visual cycle, and neurotransmitter transmission. Retinal cells, particularly RPECs and photoreceptors, require precisely regulated iron levels to maintain their structure and function. In the retina, iron primarily enters cells through the transferrin bound iron pathway. Additionally, the retina also possesses non transferrin bound iron import pathways mediated by molecules such as *Scara5*, *Zip8*, and *Zip14*. Once internalized, iron is reduced from Fe^3+^ to Fe^2+^ by the ferric reductase *STEAP3* within endosomes and subsequently transported into the cytoplasm via *DMT1*. Unused or unstored iron is exported by *FPN*, with its output regulated by hepcidin [116]. Patients with DM often exhibit iron overload in multiple organs, a condition which is related to various diabetes-related complications, including DN and DC. The frequent accumulation of iron in the retina and associated metabolic disorders are significant factors contributing to an increased susceptibility to ferroptosis in retina cells, and is related to the progress of DR.

Clinical studies indicate that serum iron indicators, such as serum iron and ferritin, are associated with the risk of DR occurrence. A study utilizing data from the National Health and Nutrition Examination Survey involving 5321 participants found a correlation between serum iron levels and the incidence of DR [117]. Another study found that patients with diabetes mellitus macular edema have elevated serum ferritin levels. Ferritin, as an indicator of the body’s iron reserves, is also an inflammatory marker for various neurodegenerative diseases. Its elevation may reflect iron-induced oxidative stress and inflammation [118]. The iron content level in the vitreous of patients with proliferative diabetic retinopathy (PDR) is 2.5 times higher than that in normal healthy human populations [119]. Similar iron accumulation was found in postmortem retinal samples of patients with DM [43]. This response indicates the presence of localized iron overload in the eyes of DR patients. Furthermore, iron accumulation in the retina of DR has been further demonstrated in animal experiments. For instance, in both STZ-induced T1DM and leptin receptor-deficient (*db*/*db*) T2DM model mice, abnormal iron deposition of was observed in the retina [43]. If DM is induced in a hereditary iron overload model mouse (*HFE* knockout mouse), it accelerates the death rate of retinal neuronal cells, expands the scope of vascular damage, and exacerbates the disruption and leakage of the BRB [43]. The hereditary hemochromatosis mouse model (hemojuvelin *Hjv* gene knockout, *Hjv*^−/−^ mice) is also a systemic iron overload model. In this model, even without inducing DM, the model mice exhibited pathological changes similar to DR, such as disrupted angiogenesis, vascular leakage, and reactive gliosis, suggesting that iron overload may be a direct causative factor of DR [120].

**Figure 1:**
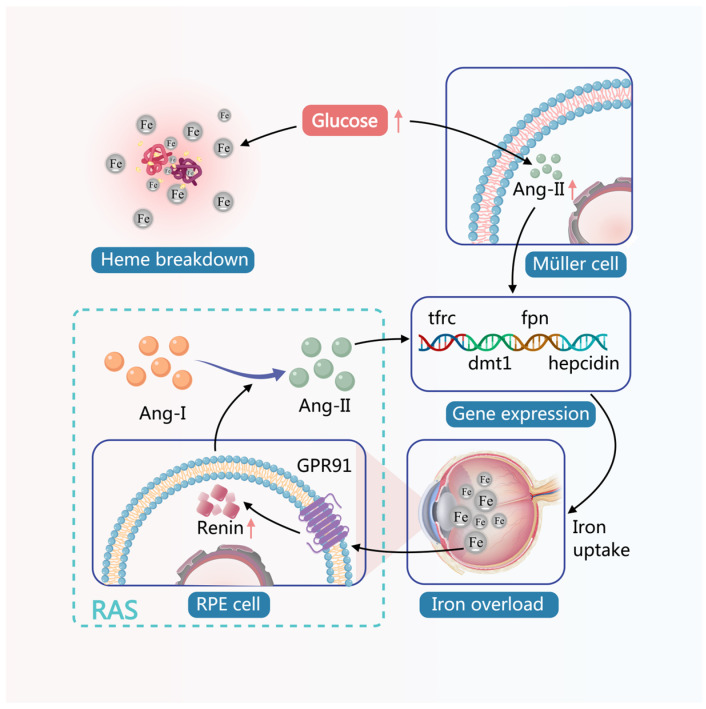
Retinal Iron Overload in Diabetic Retinopathy. Hyperglycemia initiates a vicious cycle in the retina involving iron overload and activation of the local renin-angiotensin system (RAS), ultimately leading to retinal damage. The pathological process begins with hyperglycemia, which directly induces heme breakdown, increasing free iron levels. Concurrently, high glucose stimulates initial expression of Angiotensin-II (Ang-II) in Müller cells. This initial surge of Ang-II upregulates key iron metabolism genes (e.g., tfrc, dmt1), further enhancing cellular iron import. The resulting retinal iron overload then activates the G-protein coupled receptor 91 (GPR91) signaling pathway in retinal pigment epithelial cells (RPECs), promoting renin expression. Increased renin activity catalyzes the RAS cascade, leading to a substantial secondary production of Ang-II within the retinal tissue. This newly generated Ang-II, in turn, exacerbates iron dysregulation, creating a self-amplifying local vicious cycle between Ang-II and iron overload that operates across Müller and RPECs, ultimately accelerating retinal injury.

In the mechanism through which DM causes an imbalance in retinal iron metabolism, hyperglycemia can first directly induce the destruction of heme molecules, leading to an increase in free iron [121]; Secondly, the hyperglycemic environment can significantly induce the expression of Ang-II in Müller cells [122,123], Ang-II may stimulate the expression of iron metabolism-related genes, including *tfrc*, *dmt1*, *fpn*, and *hepcidin*, thereby enhancing cellular iron uptake [124]. Conversely, iron overload in the retina activates the G protein-coupled receptor 91 signaling pathway, enhancing the expression of renin in RPECs [43]. The function of renin is to cleave angiotensinogen into angiotensin-I (Ang-I), which is subsequently converted into Ang-II by angiotensin-converting enzyme. Therefore, the increase in retinal renin inevitably leads to elevated Ang-II levels, which in turn further induces retinal iron overload, forming a vicious cycle between the renin-angiotensin system and iron overload, as shown in Figure 1.

Another study found that neither *db*/*db* mice nor iron-overloaded mice injected with iron dextran exhibited excessive retinal iron before 20 weeks of age, but they did show overexpression of ferritin. However, after inducing BRB breakdown through freeze-stripping, both types of mice demonstrated significant iron accumulation in the retina. This suggests that the BRB protects the retina from excessive iron during the early stages of DR progression, while the overexpression of ferritin prior to iron accumulation may prepare the retina for excessive circulating iron input in the later stages [125].

### 3.2. GPX4-Dependent Ferroptosis Regulation Signaling and DR

**Figure 2:**
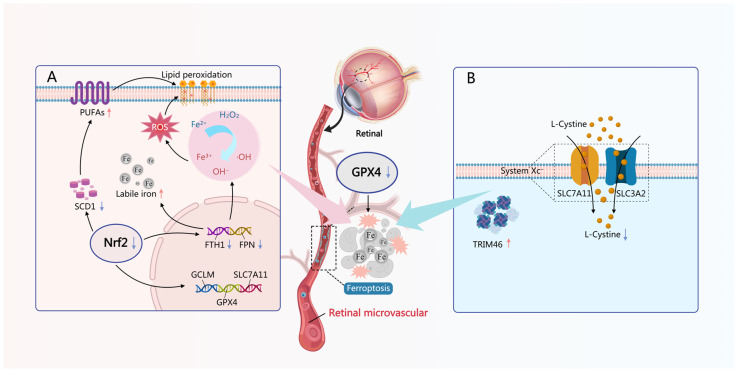
GPX4-dependent ferroptosis signaling in DR. (**A**) Nrf2 inhibition downregulates the expression of GPX4, GCLM, SCD1, and iron metabolism regulators (FTH1, FPN), leading to increased labile iron (Fe^2+^) and peroxidizable PUFAs—collectively fostering an environment prone to labile iron pool (Fe^2+^) expansion and lipid peroxidation. (**B**) High glucose upregulates the E3 ubiquitin ligase TRIM46, promoting ubiquitination and degradation of GPX4 protein. Together, these convergent pathways lead to a critical failure in lipid peroxide repair, executing ferroptosis and driving the pathogenesis of diabetic retinopathy.

As mentioned earlier, GPX4, as a selenoprotein GSH peroxidase, serves as the primary defense system for cells against oxidative stress and ferroptosis. Studies have found that GPX4 is expressed in various retinal cell types, including RPECs, retinal neurons, glial cells, and endothelial cells [126], and the SNP (*rs*713041) of the *GPX4* gene was found to be associated with the risk of developing PDR in patients with T1DM [127]. *GPX4* conditional knockout mice exhibit abnormal differentiation and significant loss of RPECs during development stage [128]. Correspondingly, the depletion of GSH also leads to premature aging of RPECs, manifested as cell growth arrest and increased expression of senescence-associated genes [129]. This indicates that the GPX4-GSH-dependent antioxidant system is crucial for the development and survival of retinal cells. Under DM conditions, elevated levels of ROS lead to membrane lipid peroxidation, generating harmful lipid peroxides and ferroptosis of retinal cells [130]. Multiple studies have demonstrated that the expression and activity of GPX4 are significantly downregulated in DR patients and DM animal models, accompanied by elevated levels of ferroptosis markers such as lipid peroxidation products (e.g., malondialdehyde, MDA) and iron ions [46,131,132]. Firstly, under high glucose (HG) induction, the expression of *TRIM46* in human RMECs (HRMECs) increases, accelerating the degradation of *GPX4* by promoting its ubiquitination. This results in reduced GPX4 levels, which subsequently induces ferroptosis and inhibits cell growth [133]. Secondly, under HG conditions, the Nrf2 is inhibited, thereby affecting *GPX4* expression [134]. Furthermore, the GSH levels in the retinas of diabetic rats were significantly diminished, resulting in an inadequate substrate for GPX4 and consequently limiting its antioxidant capabilities. For example, in STZ treated rats, the levels of *SLC7A11* and the activity of the system XC^−^ in the retina were diminished. Since system XC^−^ is a cystine-glutamate antiporter, its impaired function led to reduced cystine uptake. This, in turn, limited the availability of cysteine, the rate-limiting substrate for GSH synthesis, resulting in a 23% decrease in intracellular total GSH content. Concurrently, the dysfunction of the antiporter also altered glutamate flux, explaining the observed reduction in total L-[^3^H]-glutamic acid uptake. These metabolic disruptions were accompanied by an increase in oxidative stress markers within the retina [135], as shown in Figure 2.

### 3.3. P53-Dependent Ferroptosis Regulation Signaling and DR

**Figure 3:**
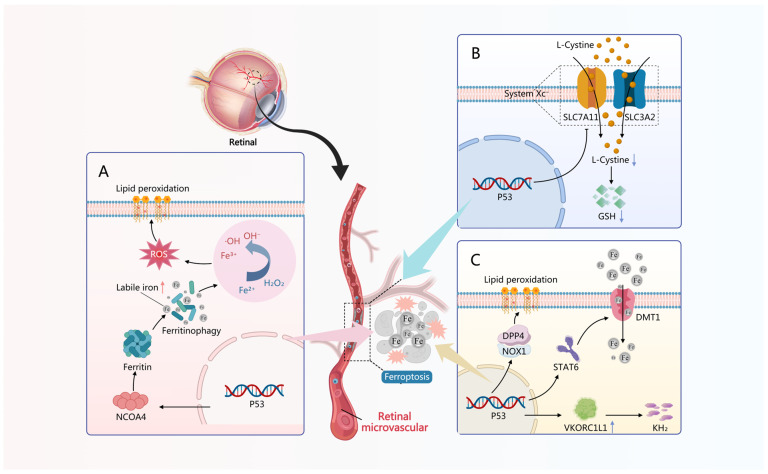
p53-dependent ferroptosis signaling in DR. Elevated p53 promotes ferroptosis in retinal microvascular endothelial cells through a coordinated multi-pronged strategy: (**A**) Iron Dysregulation: p53 activates ferritinophagy via NCOA4, increasing labile iron pools and Fenton reaction-derived ROS. (**B**) Antioxidant Inhibition: p53 represses SLC7A11 to impair System Xc^−^ function, depleting glutathione (GSH) and invalidating the GPX4 system. (**C**) Pro-Peroxidation Mechanisms: p53 drives lipid peroxidation by facilitating the NOX1-DPP4 interaction, enhancing iron uptake via the STAT6-DMT1 axis, and suppressing the anti-ferroptotic VKORC1L1/vitamin K hydroquinone pathway. These converging mechanisms lead to GPX4 depletion, lethal lipid ROS accumulation, and retinal microvascular damage in diabetic retinopathy.

Bioinformatics research utilizing disease expression profile data has revealed that *TP53* (the coding gene for p53) is one of the core characteristic genes of DR and may serve as a diagnostic marker gene for DR [136,137,138,139,140]. Through single-cell RNA sequencing of retinal tissues from *db*/*db* mice, seven major cell clusters were identified, with *TP53* highly expressed in the endothelial cell cluster. Its expression level was also significantly elevated compared to healthy control mice, accompanied by increased senescence-associated β-galactosidase activity, upregulation of senescence-related genes, and elevated expression of senescence-associated secretory phenotype factors. These findings suggest that the senescent phenotype observed in the retina under DM conditions may be associated with p53, with vascular endothelial cells potentially being the primary contributors [141]. In animal experiments, the protein level of p53 was significantly elevated in retinal endothelial cells of STZ-induced DM mice, accompanied by a marked decrease in the antioxidant factors *Slc7a11* and *Gpx4*, suggesting an increase in p53-dependent ferroptosis in RMECs under DM conditions [141,142]. In vitro experiments, cultured HRMECs were treated with advanced glycation end products to simulate DR. It was similarly observed that elevated p53 expression in the cells was accompanied by decreased expression levels of *SLC7A11* and *GPX4*, as well as increased lipid-ROS levels, iron overload and promoting ferroptosis [143]. The same phenomenon was also identified in HRMECs cultured under HG conditions [144], as shown in Figure 3.

### 3.4. The Regulation of Ferroptosis by Non-Coding RNAs in DR

Non-coding RNAs, particularly lncRNAs and miRNAs, regulate ferroptosis in DR through various molecular mechanisms, including influencing iron metabolism, lipid peroxidation, antioxidant defense systems, and related signaling pathways (such as m6A, *Nrf2*, *p53*, and PI3K/Akt). These studies not only deepen our understanding of the pathogenesis of DR but also provide new directions for developing ncRNA-based diagnostic biomarkers and therapeutic strategies for DR. In high-glucose cultured *Homo sapiens* retinal vascular endothelial cells, the lncRNA zinc finger antisense 1 (*ZFAS1*) is upregulated, and suppression of *ZFAS1* alleviates HG-induced elevation of ROS levels and ferroptosis. Further validation revealed that *ZFAS1* may act as a competitive endogenous RNA by competitively binding with miR-7-5p, thereby relieving the miRNA’s inhibitory effect on the expression of its downstream molecule long-chain *ACSL4*. This leads to sustained upregulation of *ACSL4* expression, subsequently increasing the production of lipid peroxidation substrates PL-PUFAs and enhancing cellular susceptibility to ferroptosis [145]. There are some other lncRNAs which, although not directly demonstrated to contribute to ferroptosis in retinal cells during DR, have been found to potentially participate in retinal angiogenesis in DR, such as lncRNA *HOTAIR*, lncRNA *RP11-502I4.3*, and lncRNA *MEG3* [146,147,148].

From the research on miRNAs, bioinformatics analysis and miRNA prediction based on GEO expression profile data indicate that several miRNAs such as hsa-miR-873-5p may be involved in the regulation of key ferroptosis-related genes in retinal cells during DR [138]. In animal- and cell-based experimental studies, it was found that miR-509-3p can directly target the mitochondrial aspartate/glutamate carrier *SLC25A13* in HRMECs, inhibiting *SLC25A13*-dependent ferroptosis, thereby protecting RMECs from damage under HG conditions [149]. *miR-214-3p* has also been found to specifically target *P53*, thereby inhibiting ferroptosis in HRMECs during DR by regulating the *P53*/*SLC7A11*/*GPX4* axis [144]. Mesenchymal stem cell (MSC)-derived exosomes can alleviate damage to the BRB in DR rats by reducing ferroptosis. Further studies revealed that miR-125b-5p in MSC exosomes reverses ferroptosis in HRMECs under DR conditions by targeting and downregulating P53 expression [143]. The amino acid transporter *SLC1A5* is a known ferroptosis promoter that functions by transporting glutamine into the cells, where it is converted to glutamate by glutaminase. Glutamate then enters mitochondria and is transformed into α-ketoglutaric acid under the catalysis of glutamate oxaloacetate transaminase and glutamate dehydrogenase 1 (*GLUD1*). This process sustains cellular lipid synthesis but simultaneously generates substantial ROS, creating a highly oxidative environment that influences cellular susceptibility to ferroptosis, indicating that *SLC1A5* is one of the important targets regulating ferroptosis [150,151,152]. The study revealed that miR-338-3p facilitates the inhibition and degradation of the enzyme by targeting the 3′ UTR of SLC1A5 in RPECs. Additionally, HG culture conditions influence ferroptosis in RPECs by modulating the production of miR-338-3p, indicating another potential mechanism in the progression of DR [153].

circRNA is a special type of non-coding RNA that forms a covalently closed circular structure, which is not easily degraded and exhibits stable expression. Although existing literature provides limited direct elucidation on the detailed mechanisms of circRNA in ferroptosis during DR, it is generally believed that circRNA can regulate gene expression by acting as an miRNA sponge or through other ways, thereby influencing cell death, including ferroptosis [154]. Several circRNAs have been demonstrated to be associated with DR. For instance, circular DNA methyltransferase 3B (*circDNMT3B*) acts as a sponge for miR-20b-5p, promoting retinal vascular dysfunction in DR [155]. Circular leucine-rich repeat kinase 3 (*CircHIPK3*) mediates retinal vascular dysfunction through the *CircHIPK3-miR-30a-3p*-*VEGFC*/Wnt signaling pathway protein 2/frizzled protein 4 (*FZD4*) network in DR [156]. Another study revealed that in the HG-treated *h*RMEPCs Adult Retinal Pigment Epithelial cell line-19, the expression of *circ-PSEN1* was upregulated; inhibiting *circ-PSEN1* alleviated HG-induced ferroptosis in the cells. Further investigation indicated that *circ-PSEN1* functions as a sponge for miR-200b-3p, counteracting the inhibitory effect of miR-200b-3p on the expression of the actin-binding protein-2 (*CFL2*), thereby promoting *CFL2*-dependent ferroptosis. Nonetheless, the role of *CFL2* in ferroptosis necessitates further investigation [157].

### 3.5. The Role of the Autophagy-Lysosome and Ferroptosis Cross-Regulatory Pathway in DR

Autophagy is a self-degradative pathway for intracellular substance degradation and recycling. It degrades various biomacromolecules and organelles through the lysosomal pathway to adapt to energy supply and maintain cellular homeostasis. In recent years, the intricate crosstalk between autophagy and ferroptosis has garnered widespread attention. Initially, ferroptosis was considered a form of cell death independent of autophagy. However, mounting evidence suggests that autophagy plays a pivotal role in the occurrence and regulation of ferroptosis, which can be either promotive or inhibitory depending on the cellular context and disease type [158,159,160]. Autophagy plays a dual role in DR, promoting cell survival while potentially causing cellular damage when excessive [161]. Numerous studies have demonstrated that autophagy dysfunction is an early event in the pathogenesis of DR. For instance, HG-induced autophagy defects in Müller cells exacerbate DR progression, whereas treatment with the autophagy enhancer rapamycin improves autophagic flux and lysosomal proteolytic activity in Müller cells, while preventing excessive VEGF release, thereby ameliorating the microvascular lesions in DR [162]. Furthermore, downregulation of high mobility group box 1 protein (HMGB1) protects RPECs from DR damage through the autophagy-lysosome pathway [163]. miR-1273g-3p was also found to be involved in the development of DR through the regulation of the autophagy-lysosome pathway [164].

As previously mentioned, ferritinophagy, as a specialized autophagy pathway, plays a critical role in cellular iron overload and ferroptosis. In the context of DR, mitochondrial dysfunction under HG conditions, mitophagy, and ferritinophagy may be associated with ferroptosis [158]. A study found that the key regulatory factor of ferritinophagy, *NCOA4*, was upregulated in 661W cells cultured under HG conditions and in RPECs of DM mice, suggesting that ferritinophagy may be involved in retinal cell ferroptosis in DR [131]. Furthermore, excessive mitophagy may lead to dysregulation of iron metabolism and the occurrence of ferroptosis, thereby promoting the progression of DR. A study utilized differential expression analysis from the GSE146615 expression profile dataset to identify DEGs associated with ferroptosis in DR. A total of 8 DEGs were identified, among which autophagy related genes such as *BECN1*, *HERC2*, *ATG7*, and *BCAT2* may serve as potential biomarkers for DR. This suggests that these genes might influence the onset and progression of DR through the regulation of ferritinophagy [165]. Analysis of single-cell sequencing data from DR retinal tissues also identified 63 ferroptosis-related differentially expressed marker genes that were significantly enriched in peroxidase activity, ferroptosis, mitophagy, and macroautophagy [166]. Sestrin2 (*SESN2*) is a key regulatory factor in the stress response, primarily involved in mitochondrial stress adaptation. It plays crucial roles in the integrated stress response, mitochondrial biogenesis, and mitophagy, serving as a regulatory hub between mitochondrial stress and autophagy [167]. HG and STZ-treated ARPE-19 cells and C57BL/6 mice were utilized to establish in vitro and in vivo models of DR. The results indicated a reduction in *SESN2* expression, an increase in the apoptosis rate, activation of ER stress, a decrease in autophagy levels, and an elevation in ferroptosis levels. However, the overexpression of *SESN2* improved cell viability, decreased apoptosis and ferroptosis, and stimulated mitophagy, thereby offering protection against HG-induced RPECs damage. The protective effects of *SESN2* overexpression were negated by the use of the ferroptosis activator erastin or the autophagy inhibitor 3-MA. These findings suggest that *SESN2* also acts as a regulatory hub for mitophagy and ferroptosis in DR [168].

The interplay between autophagy and ferroptosis in DR extends beyond ferritinophagy and mitophagy; key regulatory proteins of ferroptosis can also be influenced by autophagy. During the initial stages of DM, a neurodegenerative factor in the vitreous, glia maturation factor-β (*GMF-β*), shows increased expression. Under hyperglycemic conditions, the vitreous secretes significant quantities of *GMF-β* protein, which expels the ATPase *ATP6V1A* from the lysosomes in RPECs, hindering its assembly and causing lysosomal alkalinization. The protein *ACSL4* can be targeted by the chaperone-mediated autophagy receptor *HSC70* and subsequently broken down in lysosomes. However, with HG levels, the autophagy-lysosomal dysfunction resulting from elevated *GMF-β* levels hampers the efficient degradation of ACSL4 protein. The buildup of *ACSL4* facilitates the generation of lethal lipids, ultimately triggering ferroptosis in RPECs [169].

## 4. Ferroptosis-Mediated Cell Type-Specific Damage in DR

### 4.1. RMECs

RMEC injury and death constitute a critical component of the early pathological changes in DR, serving as a hallmark event in DR progression that leads to BRB disruption and increased vascular leakage [170]. In the early stages of DM, there is an increase in retinal leukocyte stasis accompanied by upregulation of retinal leukocyte adhesion molecules (such as *ICAM-1*, *VCAM-1*) [171]. These inflammatory cells and molecules interact with RMECs, leading to disruption of the BRB and vascular leakage [172,173]. Moreover, inflammatory cells such as macrophages and microglia also play a significant role in the occurrence and progression of DR. Their crosstalk with endothelial cells regulates DM-induced retinal vascular dysfunction by remodeling the inflammatory microenvironment [174]. In addition to the role of inflammation, HG-induced mitochondrial dysfunction and oxidative stress are significantly increased in DR, leading to the translocation of the pro-apoptotic protein Bax and the release of cytochrome c, which subsequently activates caspases and ultimately results in apoptosis of RMECs [175]. Although oxidative stress is also an initiating factor of ferroptosis, the execution mechanism of ferroptosis is entirely different from apoptosis. However, as a complement to apoptosis, ferroptosis further exacerbates the loss and dysfunction of RMECs in DR, this in turn leads to impaired intercellular connections and disruption of the BRB’s integrity, manifested as increased vascular permeability and leakage [133]. During this process, hemorrhage caused by blood leakage results in hemoglobin degradation, with free iron further accumulating in the retina, exacerbating retinal iron overload. Consequently, this further increases the susceptibility of retinal cells to ferroptosis.

**Figure 4:**
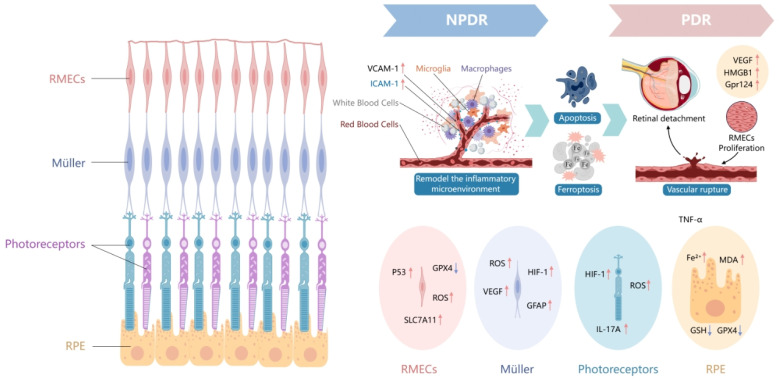
Ferroptosis-mediated cell type-specific damage in DR. Retinal Microvascular Endothelial Cells (RMECs): Early-stage alterations include inflammation (upregulation of ICAM-1/VCAM-1), mitochondrial apoptosis (Bax/cytochrome c), and ferroptosis, leading to the breakdown of the blood-retinal barrier (BRB). In the late stage, hypoxia drives aberrant proliferation and migration (mediated by VEGF/HMGB1/Gpr124), resulting in pathological neovascularization. Key genes (e.g., P53, HIF1A) and signaling pathways (e.g., HIF-1, FoxO, MAPK) are involved in these processes. Müller Cells: These cells undergo gliosis (increased GFAP expression) and overproduce factors like VEGF, thereby amplifying inflammatory responses, promoting vascular leakage, and contributing to neovascularization. Their function is critically regulated by HIF-1. Retinal Pigment Epithelial Cells (RPECs): These cells face high glucose-induced mitochondrial dysfunction, oxidative stress, and ferroptosis (evidenced by elevated Fe^2+^/MDA and inhibited GSH/GPX4). Furthermore, inflammatory cytokines (e.g., TNF-α) can upregulate mitophagy, exacerbating cell death. Photoreceptor Cells: These cells exhibit metabolic disturbances, increased ROS production, and undergo both apoptosis and ferroptosis. Their function is influenced by factors such as HIF-1α, Wnt signaling, and IL-17A.

In the stage of PDR, it primarily involves the abnormal proliferation of RMECs and the formation of new blood vessels, which is the main cause of severe vision loss [176]. Hypoxia-induced VEGF expression promotes the proliferation and migration of RMECs, forming fragile neovascularization that is highly prone to rupture and hemorrhage, leading to vitreous hemorrhage and retinal detachment [176]. In addition to VEGF, other factors such as *HMGB1* and *Gpr124* are also involved in regulating the proliferation and migration of RMECs, promoting the formation of PDR [177,178]. Although there is currently no experimental evidence demonstrating the direct role of ferroptosis in the proliferation and migration of RMECs in PDR, some bioinformatics analyses have suggested its potential involvement. For instance, in the studies analyzing the expression profiles of datasets GSE60436 and GSE94019 from the GEO database, 21 upregulated and 9 downregulated ferroptosis-related DEGs were identified, including 10 key target genes (*p53*, *TXN*, *PTEN*, *SLC2A1*, *HMOX1*, *PRKAA1*, *ATG7*, *HIF1A*, *TGFBR1*, and *IL1B*) that were enriched in biological processes related to oxidative stress and hypoxia response in PDR. Furthermore, HIF-1, FoxO, and MAPK signaling pathways may represent the primary mechanisms influencing ferroptosis in PDR [137]. In another analysis of GEO expression profile data, it was found that expression of M2 macrophage characteristic genes was significantly higher in fibrovascular membrane samples from PDR patients compared to control retinas. Three hub intersection genes including *TP53*, *HMOX1*, and *PPARA* were identified through analysis of the DEGs enriched in M2 macrophage-related and ferroptosis-related genes. qRT-PCR validation showed that *HMOX1* expression was significantly elevated in the retinas of oxygen-induced retinopathy mouse models compared to controls. Single-cell analysis confirmed *HMOX1* localization within M2 macrophages, which was further validated by immunofluorescence staining. This study suggests that *HMOX1* expression is associated with M2 macrophage infiltration and ferroptosis, potentially playing a critical role in the pathogenesis of PDR [179], as shown in Figure 4.

### 4.2. RPECs

The retinal pigment epithelium (RPE), as the outer layer of the BRB, is crucial for maintaining retinal homeostasis. Its functions include the transport of nutrients and ions, light absorption, retinoid recycling, as well as phagocytosis of cone/rod cells’ outer segments [180]. RPECs play a crucial role in the development and progression of DR. Their structural changes and functional alterations constitute an important component of early pathological changes in DR, and may even occur before clinical symptoms manifest [181,182]. In DM patients without DR changes, alterations have been observed in the thickness, volume, and reflectivity of the RPE-photoreceptor complex, including disruptions of the external limiting membrane, photoreceptor ellipsoid zone, and RPE layer [183]. A HG environment can cause damage to RPECs, impair their normal cellular connections, thereby disrupting the integrity of BRB, leading to increased vascular permeability and leakage [184]. In the DM rat model, activation of the ROCK-1 pathway was found to mediate vesicle formation (blebbing) in RPE and endothelial cells, which is associated with retinal capillary occlusion and BRB disruption in DR [185]. Additionally, HG increase oxidative stress levels and promote apoptosis in RPECs [186,187]. In DR, inflammatory factors such as TNF-α can upregulate mitochondrial autophagy in RPE cells, thereby exacerbating their death [188]. In addition, HG may also promote the abnormal proliferation and migration of RPECs, which is associated with the progression of DR [186]. The RPECs also work closely with photoreceptor cells to maintain the light-sensing function of the retina. Under DM conditions, the supportive function of the RPE for photoreceptor cells is impaired, leading to damage and dysfunction of photoreceptor cells. Patients with DM exhibit decreased dark adaptation ability, which is associated with dysfunction of both the RPE and photoreceptor cells [189]. Therefore, dysfunction of the RPE not only affects the function of BRB but also exacerbates damage to the neural retina, leading to degeneration of photoreceptor cells and neurons.

Ferroptosis of RPECs is a hallmark feature of DR, particularly in the late-stage age-related macular degeneration [190,191]. Studies have shown that HG can significantly increase ROS levels in RPECs [192], leading to accumulation of iron-dependent lipid peroxidation products, which represents the core biochemical hallmark of ferroptosis [191]. Study have also shown that in HG-treated RPECs, levels of lipid peroxidation products such as Fe^2+^ and MDA increase, while GSH level decrease and *GPX4* activity is inhibited [157]. These are key markers of ferroptosis occurrence. Ferroptosis not only affects the quantity and function of RPECs, but also participates in the degeneration of retinal photoreceptor cells and the dysfunction of RMECs, leading to microvascular leakage and retinal damage [131].

### 4.3. Photoreceptor Cells

Although DR has traditionally been considered a microvascular disease, mounting evidence suggests that retinal neurons, particularly photoreceptor cells, play a pivotal role in the onset and progression of DR [189]. Clinical studies have shown that in DR patients, photoreceptor cell dysfunction may occur earlier than the onset of vascular lesions, and DM-related photoreceptor damage may promote the development of retinal microvascular diseases [193]. Self-adaptive optical imaging technology can be used to evaluate the differences in photoreceptor cell morphology between DR patients and healthy controls [194]. Dark adaptation tests also revealed that RPE dysfunction and impairments in rod and cone photoreceptor cells in DR patients progressively worsen with the advancement of DR severity [195]. First, photoreceptor cells are one of the primary sources of retinal oxidative stress in DR [196]. Under hyperglycemic conditions, the metabolic activity of photoreceptor cells is often altered, generating excessive ROS that damages cellular structures and functions, which may be associated with the roles of hypoxia-inducible factor-1α (HIF-1α) and Wnt signaling pathways [197]. More strikingly, the degeneration of photoreceptor cells in opsin gene knockout (*opsin*^−/−^) mice or the experimental destruction of photoreceptor cells with iodoacetic acid could both significantly suppress the generation of superoxide in the retina induced by DM [196]. Secondly, in the context of DM, photoreceptor cells play a certain role in the release of inflammatory mediators, exacerbating retinitis [196]. *IL-17A* is upregulated in the retina of DM and may participate in the development of DR by affecting photoreceptor cell apoptosis [198]. Finally, as a type of neuron, the death and dysfunction of photoreceptor cells caused by HG environment directly lead to neuronal degeneration, subsequently affecting visual signal processing [199]. Ferroptosis, as a form of programmed cell death, is also involved in the degeneration of photoreceptor cells in DR. For instance, HG can induce oxidative stress and ferroptosis in cultured *661W* cells (mouse retinal photoreceptor cells). Ferroptosis in photoreceptor cells has also been detected in the retinas of DM mice, and this process can be reversed by the ferroptosis inhibitor ferrostatin-1 (Fer-1) [131].

### 4.4. Müller Cells

Müller cells, as the primary glial cells in the retina (constituting 90% of retinal glial cells), are crucial for maintaining retinal homeostasis. These cells are radially distributed throughout the retina, providing structural support for retinal neurons. They participate in the recycling of neurotransmitters, such as glutamate, thereby preventing excitotoxicity. Simultaneously, Müller cells also supply metabolic support to retinal neurons and regulate ion and water balance in the retina, maintaining the stability of the extracellular environment [200,201,202]. Secondly, Müller cells interact with retinal blood vessels, regulating vascular permeability and maintaining the integrity of the BRB. An HG environment induces Müller cells to produce excessive ROS and oxidative stress, which subsequently damages various biomolecules within the cells, including proteins, lipids, and DNA, exacerbating Müller cell death and dysfunction [203]. Studies indicates that HG can promote the activation of Müller cells and the release of pro-inflammatory cytokines, such as IL-1β, IL-6, and TNF-α. These cytokines further activate other immune cells in the retina, such as microglia, forming an inflammatory cascade response that damages retinal neurons and blood vessels [174,188,201,204]. During the progression of DR, Müller cells overexpress VEGF, promoting the formation of retinal neovascularization [205]. HIF-1 is considered to play a key regulatory role in Müller cells for retinitis and neovascularization [206]. Under DM conditions, Müller cells may be unable to effectively clear extracellular glutamate, leading to neuronal excitotoxicity [200]. Moreover, the inflammatory response and oxidative stress involving Müller cells can also directly damage neurons, leading to retinal neurodegeneration [207]. In the late stage of DR, Müller cells undergo gliosis, manifested by increased expression of glial fibrillary acidic protein (*GFAP*) [208]. This proliferative response may lead to alterations in the retinal structure and facilitate the occurrence of tractional retinal detachment [209].

Recent studies have observed the presence of ferroptosis in Müller cells during the progression of DR. For example, HG treatment can lead to decreased levels of antioxidant function markers such as GSH, SOD, and MDA in mouse Müller cells, accompanied by an increase in ferroptosis markers. This may be related to the roles of aquaporin-4 and transient receptor potential cation channel subfamily V member 4 [210]. HG also reduced nuclear *Nrf2* expression in Müller cells, indicating that the Nrf2 pathway plays a role in ferroptosis of Müller cells under DR conditions [134,211]. In summary, ferroptosis in Müller cells is a critical link in the progression of DR, playing a pivotal role in promoting both the dysfunction of Müller cells and the pathological changes in the retina.

## 5. Translational Research on Ferroptosis and DR Clinicopathology

Current research on ferroptosis in DR based on human tissue samples is extremely limited. Apart from previous studies that identified increased iron levels in retinal tissues from diabetic patients, a recent study from a Chinese research team utilized reverse RT-qPCR to examine ferroptosis-related marker genes in fibrovascular membranes from patients with PDR undergoing vitrectomy, as well as in epiretinal membranes from macular hole or epiretinal membrane patients who were used as controls. These genes included *GPX4*, *ACSL4*, *FTH1*, *TfR*, and cyclooxygenase-2. The results demonstrated significantly altered expression of these ferroptosis-related genes in PDR retinal tissues, indicating that the ferroptosis pathway is activated at least during the advanced stages of DR [44]. A bioinformatics study from China employed two human DR retinal tissue expression profile datasets, GSE60436 and GSE102485, identifying a total of 19 ferroptosis-related genes among the differentially expressed genes and 5 core genes were screening out including *CAV1*, *TLR4*, *TP53*, *IL-1B*, and *HMOX1*. The expression of these genes was subsequently validated through in vitro study and confirmed to be associated with ferroptosis and cellular functions [140].

## 6. Therapeutic Prospects in DR by Targeting Ferroptosis

### 6.1. Inhibiting Lipid Peroxidation

Among commonly used ferroptosis inhibitors, Fer-1 can directly scavenge free radicals generated during lipid peroxidation, such as lipid peroxidation radicals and alkoxy radicals, thereby interrupting the chain reaction of lipid peroxidation [212]. In several DR animal models, Fer-1 has been shown to inhibit retinal iron overload, enhance the expression and activity of *GPX4*, increase the reduced state of glutathione, improve the antioxidant capacity of retinal cells, alleviate ferroptosis, and ultimately ameliorate retinal microangiopathy in DR [44,46,131]. Liproxstatin-1 (LX-1) is also a selective ferroptosis inhibitor that can scavenge lipid radicals and regulate the redox state and activity of iron ions within lysosomes. It blocks the initiation of the lipid peroxidation chain reaction, thereby inhibiting the accumulation of iron-dependent lipid hydroperoxides and significantly reducing intracellular ROS levels, while maintaining or even restoring the levels and activity of *GPX4* [213,214,215]. In vivo study found that LX-1 treatment effectively prevented early DR lesions and maintained normal visual function in model rats [169]. Moreover, given the role of the NOX family in promoting cellular oxidative stress and ferroptosis, Nox2 inhibitors such as apocynin and EHop-016 have been demonstrated in vitro to ameliorate ferroptosis markers, mitochondrial damage, and cell death in HG-cultured HRMECs [130].

### 6.2. Reducing Iron Overload

The accumulation of iron ions acts as a direct trigger for ferroptosis. Chelating excess iron or regulating iron homeostasis can prevent the occurrence of ferroptosis. Although current studies have not directly mentioned intervention research with iron chelators in DR animal models, certain iron chelators, such as deferoxamine, have been proven effective in in vitro models of DR. For instance, treatment with 50 μM deferoxamine in HRMECs cultured under HG conditions reduced ferroptosis markers and alleviated mitochondrial damage and cell death, suggesting reducing iron overload through iron chelators is also a potential therapeutic direction for DR [130].

### 6.3. Nrf2 Activators

The transcription factor Nrf2 serves as a central regulator of cellular antioxidant responses and a potent endogenous inhibitor of ferroptosis. By orchestrating the expression of a network of cytoprotective genes (e.g., SLC7A11, GPX4, FTH1), Nrf2 activation simultaneously fortifies multiple defensive fronts against ferroptosis, including glutathione synthesis, iron homeostasis, and lipid metabolism. Given this pivotal role, pharmacological activation of Nrf2 has emerged as a distinct and promising therapeutic strategy for DR, aiming to systemically bolster retinal cellular resilience against high glucose-induced ferroptotic damage. Several compounds, particularly from natural sources, have demonstrated significant efficacy in DR models primarily through Nrf2-dependent mechanisms. For instance, Corilagin (COR) is a water-soluble tannin derived from plants that exhibits multiple biological activities. It has been shown preventive and therapeutic effects on various cardiovascular and cerebrovascular diseases, such as hypertension, atherosclerosis, stroke, congestive heart failure, and ischemic cardiomyopathy. The mechanism of its action is linked to its ability to alleviate oxidative stress. Research has indicated that COR activates the Nrf2-dependent antioxidant signaling pathway both in vivo and in vitro, preventing HG-induced changes in retinal morphology and biochemical parameters, including lipid peroxidation, iron deposition, and ferroptosis, while also mitigating damage to retinal tight junction proteins [216]. Similarly, Resveratrol (RSV) exemplifies a multi-target approach centered on Nrf2 activation. It inhibits ferroptosis in retinal Müller cells via the Nrf2/GPx4/PTGS2 pathway [134]. Furthermore, RSV also protects against DR by activating the SIRT1/HMGB1 pathway in HRCECs, which concurrently suppresses retinal inflammation, angiogenesis, and oxidative stress, thereby alleviating ferroptosis and microvascular lesions [217]. Carnosic acid (CA) is also a natural anti-inflammatory and antioxidant compound that exhibits therapeutic potential in various types of cancers. In experimental models of neurodegenerative diseases, CA has demonstrated neuroprotective effects, primarily through the activation of the Nrf2/ARE-dependent antioxidant pathway [218]. CA has also been found to alleviate oxidative stress, inflammation, and apoptosis in the retinal tissues of STZ-induced DR mice. In vitro experiments confirmed that CA exhibits a dose-dependent enhancement of *SIRT1* expression, prevents ferroptosis in HRMECs by activating the SIRT1/p53/SLC7A11 pathway, thereby providing substantial protection against high glucose (HG)-induced HRMECs damage [219].

### 6.4. Other Natural Compounds and Metabolites with Ferroptosis-Inhibiting Potential

Beyond Nrf2 activators, a variety of other natural compounds have shown promise in mitigating ferroptosis in DR models through diverse molecular pathways. These agents highlight the multiple entry points for therapeutic intervention. For example, the metabolite of lysine, pipecolic acid, serves as an important immune regulator in plants and *Homo sapiens*. It can alleviate ferroptosis in DR by targeting the YAP-GPX4 signaling pathway, thereby improving the DR phenotype in DM models [220]. 1,8-Cineole is the main component of volatile oils in aromatic plants and possesses various pharmacological effects, including antioxidant, anti-inflammatory, and anticancer activities. Research has found that this compound can inhibit HG-induced ferroptosis in RPECs through the PPAR-γ/TXNIP pathway, while rosiglitazone, as a *PPAR-γ* activator, exhibits the same effect [221]. Isoquercetin, a flavonoid compound derived from Bidens pilosa, can also be synthesized artificially. It exhibits antioxidant, blood pressure-reducing, and anti-inflammatory properties. This compound can mitigate retinal damage in mice with DM by inhibiting *P53* [142].

### 6.5. Vitamins

Existing studies have found that the reduction in vitamin D plays a critical role in oxidative stress and vascular endothelial injury induced by DM. In vitro experiments revealed that under high-glucose conditions, 25(OH)D3 treatment significantly promoted the proliferation of hRMVECs, markedly reduced intracellular ROS/MDA levels, and upregulated GSH levels. Furthermore, 25(OH)D3 substantially decreased Fe^2+^ levels in cells while increasing the protein levels of GPX4 and SLC7A11. Further investigation demonstrated that the effects of 25(OH)D3 were associated with its downregulation of *miR-93* expression levels [222]. Vitamin K is another fat-soluble vitamin, and extensive basic and clinical research has found that different types of vitamin K have certain therapeutic effects on DM and its complications. For example, vitamin K2 has been proven to improve DM-related cognitive decline by reducing oxidative stress and neuritis [223]. In addition, vitamin K inhibits vascular calcification in DM by activating matrix Gla protein (MGP), thereby alleviating cardiovascular complications caused by DM [224]. Although there is currently no direct research evidence indicating that vitamin K supplementation has therapeutic effects on DR, recent studies have found that vitamin K is also involved in the regulation of ferroptosis. The study revealed that *VKORC1L1* is a potent ferroptosis suppressor, which protects cells from ferroptosis by generating the reduced form of *VKORC1L1* is also a direct transcriptional target of *p53*. Activation of p53 leads to downregulation of *VKORC1L1* expression, sensitizing cells to ferroptosis and thereby inhibiting tumor growth. Small-molecule inhibitors of *VKORC1L1*, such as warfarin, suppress tumor growth by promoting ferroptosis in both immunodeficient and immunocompetent mouse models [94]. This study suggests the potential value of vitamin K in DR through the regulation of ferroptosis.

## 7. Conclusions and Perspectives

Ferroptosis, a novel form of iron-dependent cell death characterized by lipid peroxidation, has increasingly attracted attention for its involvement in the onset and progression of DR. Research conducted on animal models and in vitro cell experiments has initially uncovered the crucial pathological role of ferroptosis in DR, offering new perspectives for targeted interventions. These insights also underscore the potential for developing more effective and precise treatment strategies for DR by employing various methods, such as modulating iron metabolism, suppressing lipid peroxidation, and utilizing natural compounds. Nonetheless, numerous questions persist. For example, additional mechanistic studies are required to elucidate the precise contributions and mechanistic distinctions of ferroptosis in various stages of DR (including early neurodegenerative changes, microvascular damage, and proliferative lesions) to determine the most opportune moments for intervention. The development of DR encompasses multiple pathological mechanisms, including inflammation, oxidative stress, apoptosis, and autophagy. Ferroptosis exhibits intricate cross-regulatory interactions with these processes. For instance, oxidative stress driven by mitochondrial damage creates a vicious cycle with ferroptosis [130], and autophagy is also considered to potentially participate in the induction of ferroptosis [225]. Beyond these, future investigations should also explore the interplay between ferroptosis and other pivotal pathways in DR. For instance, the role of the inflammatory cascade and specific cytokines in modulating ferroptosis warrants in-depth examination. Likewise, the crosstalk with caspase-mediated apoptotic pathways, which are known to operate in inflammatory condition requires further elucidation [226]. Critically, recent studies have firmly established ferroptosis as a key executor of cell death in DR, directly linking this pathway to the observed oxidative stress and neuronal damage. The study provides evidence that ferroptosis contributes significantly to retinal neurodegeneration, offering a molecular basis for the early neuronal dysfunction detectable by functional assessments like the mfERG [227]. Furthermore, demonstrated that mitochondrial dysfunction in diabetes serves as an upstream trigger for ferroptosis, which in turn promotes diabetic retinal neurodegeneration [45,228]. This mechanistic insight bridges the gap between the diabetic milieu, oxidative stress, and the ensuing neuronal demise, positioning ferroptosis as a central pathological mechanism within the compromised neurovascular unit. Future research needs to establish a more comprehensive mechanism network, elucidating the precise relationship between ferroptosis and other forms of cell death as well as pathological processes. Combining ferroptosis-targeted interventions with other therapeutic strategies (such as anti-inflammatory, anti-angiogenic, and antioxidant treatments) may achieve more comprehensive therapeutic effects. In mechanistic studies, further clarification is needed regarding how different cell types in the retina interact and influence each other in DR. Individual susceptibility to ferroptosis may be influenced by genetic factors.

A particular perspective that emerges from our discussion is the role of ferroptosis in the earliest stages of DR. The observed reduction in oscillatory potentials and implicit time delays in the multifocal electroretinogram, which is established functional marker of localized neuroretinal damage, may represent early hallmarks of diabetic retinal neurodegeneration (DRN) that precede microvascular damage [229,230,231]. These functional abnormalities force a fundamental reconsideration of the DR disease sequence. The critical question is no longer merely if ferroptosis occurs, but how this specific cell death pathway in retinal neurons and glial cells initiates and propels the subsequent vascular pathology. Does ferroptosis in the inner retina serve as a primary trigger, disrupting neurovascular coupling and creating a toxic milieu that accelerates endothelial dysfunction? Clarifying the temporal and mechanistic relationship between early neuronal ferroptosis and the classic microvascular signs of DR represents a major challenge and opportunity in the field. Addressing this question could enable earlier therapeutic intervention.

From a translational perspective, although several ferroptosis-related genes (such as *NOX4* and *PARP14*) have been identified, there is a lack of specific and clinically applicable ferroptosis biomarkers, particularly non-invasive or minimally invasive markers capable of reflecting localized retinal ferroptosis status during DR progressing [139,232]. The identification of characteristic biomarkers will facilitate early diagnosis, disease progression monitoring, and evaluation of the efficacy of targeted ferroptosis therapy. In addition, challenges remain in translating these biomarkers into diagnostic tools for clinical practice. Furthermore, further screening and optimization of drugs capable of selectively targeting ferroptosis in retinal cells are needed to reduce systemic side effects. Currently, nanotechnology has been applied to targeted drug delivery and has demonstrated promising prospects in the treatment of DM and its complications [233]. Therefore, nanomaterials are expected to be utilized for delivering ferroptosis regulators, enhancing the bioavailability and targeting of drugs in the retina, thereby improving therapeutic efficacy while reducing side effects [234]. Future research could also explore the polymorphisms of ferroptosis-related genes in different DM patient populations and how these polymorphisms influence the susceptibility and progression of DR. This will contribute to achieving personalized treatment for DR. Additionally, longer-term studies in animal models are needed to evaluate the long-term efficacy and potential safety issues of targeting ferroptosis interventions, laying the foundation for clinical translation. Recent studies have highlighted the potential link between gut microbiota (GM) and eye health. Evidence supports the existence of the gut-eye axis, which is implicated in the pathogenesis of various ocular disorders, including age-related macular degeneration, uveitis and DR [235]. This means that targeting the GM, through new interventions such as probiotics, prebiotics, symbionts or fecal microbiota transplantation, may be better for managing these eye diseases [236]. Current evidence also indicates that GM and their metabolites, such as short-chain fatty acids, are involved in the regulation of ferroptosis [237,238], suggesting that therapies targeting GM may influence the progression of DR through ferroptosis regulation. In addition, recent clinical data analyses have demonstrated that GLP-1 receptor agonists such as semaglutide can reduce the risk of DR, particularly in advanced stages [239,240]. Moreover, studies have shown that semaglutide influences cellular ferroptosis via the β-Klotho pathway, thereby inhibiting the progression of diabetic nephropathy. These findings indicate that GLP-1 receptor agonists might exert therapeutic effects on DR by non-specifically targeting ferroptosis, though further experimental validation is required [241]. In conclusion, ferroptosis plays a non-negligible role in DR, and gaining an in-depth understanding of its mechanisms while developing effective targeted strategies represents an important frontier in future DR research. Addressing these key questions will help open new avenues for the prevention, diagnosis, and treatment of DR.

## Data Availability

No new data were created or analyzed in this study. Data sharing is not applicable to this article.

## Abbreviations

AGER	Advanced Glycation End Receptor
ALKBH5	AlkB Homolog 5
ALOX12	Arachidonic Acid 12-Lipoxygenase
AMPK	Adenosine Monophosphate-Activated Protein Kinase
Ang-I	Angiotensin-I
Ang-II	Angiotensin-II
ARE	Antioxidant Response Element
BRB	Blood-Retinal Barrier
CA	Carnosic Acid
CFL2	Actin-Binding Protein-2 (Cofilin-2)
COR	Corilagin
CoQ10	Coenzyme Q10
CoQ10H2	Reduced Form of Coenzyme Q10
DC	Diabetic Cardiomyopathy
DM	Diabetes Mellitus
DMT1	Divalent Metal Transporter 1
DN	Diabetic Nephropathy
DNA	Deoxyribonucleic Acid
DR	Diabetic Retinopathy
Fer-1	Ferrostatin-1
FPN	Ferroportin
FSP1	Ferroptosis Suppressor Protein 1
FTH1	Ferritin Heavy Chain 1
FTO	Fat Mass and Obesity-Associated Gene
GFAP	Glial Fibrillary Acidic Protein
GPX4	Glutathione Peroxidase 4
GSH	Glutathione
GSTZ1	GSH-Transferase Zeta 1
H3K27ac	Histone H3 Lysine 27 Acetylation
HbA1c	Hemoglobin A1c
HG	High Glucose
HMGB1	High Mobility Group Box 1 Protein
HO-1	Heme Oxygenase-1
HRMECs	Human Retinal Microvascular Endothelial Cells
IRP/IRE	Iron-Regulatory Protein/Iron-Responsive Element
Keap1	Kelch-like ECH-associated Protein 1
LATS1/2	Large Tumor Suppressor Kinase 1/2
LIP	Labile Iron Pool
LX-1	Liproxstatin-1
MAGEA6	Melanoma Antigen Family A 6
MDA	Malondialdehyde
m6A	N6-Methyladenosine
ncRNA	Non-Coding RNA
NCOA4	Nuclear Receptor Coactivator 4
NOX	NADPH Oxidase
Nrf2	Nuclear Factor Erythroid 2-Related Factor 2
•OH	Hydroxyl Radical
PDR	Proliferative Diabetic Retinopathy
PI3K-Akt	Phosphatidylinositol 3-Kinase–Protein Kinase B
PUFA	Polyunsaturated Fatty Acid
RMECs	Retinal Microvascular Endothelial Cells
ROS	Reactive Oxygen Species
RPECs	Retinal Pigment Epithelial Cells
RPE	Retinal Pigment Epithelium
RSV	Resveratrol
SESN2	Sestrin2
SLC3A2	Solute Carrier Family 3 Member 2
SLC7A11	Solute Carrier Family 7 Member 11
STAT6	Signal Transducer and Activator of Transcription 6
STEAP3	Six-Transmembrane Epithelial Antigen of Prostate 3
STZ	Streptozotocin
TAZ	Transcriptional Co-activator with PDZ-Binding Motif
TfR1	Transferrin Receptor 1
T1DM	Type 1 Diabetes Mellitus
T2DM	Type 2 Diabetes Mellitus
TXNIP	Thioredoxin-Interacting Protein
VEGF	Vascular Endothelial Growth Factor
VKORC1L1	Vitamin K Epoxide Reductase Complex Subunit 1-Like 1
WNT2	Wnt Signaling Pathway Protein 2
YAP	Yes-Associated Protein
ZFAS1	Zinc Finger Antisense 1
